# Mediastinic perforation by hemodialysys catheter during the procedure. A case report.

**DOI:** 10.1186/2036-7902-4-S1-A23

**Published:** 2012-12-18

**Authors:** Maria Farré Pinilla, S Enriquez Bargalló, N Garcia Ruiz, P Espachs Biel, J Vilà Justribó, A Montero Matamala

**Affiliations:** 1 Department of Anesthesiology, Hospital Arnau de Vilanova, Lleida, Spain

**Keywords:** central venous catheters, vascular perforation, mechanical complications

## 

We present the case of a patient, 60 years old, which has been carrying multiple central venous catheters for hemodialysis. We recived consultation: the patient need a central vascular access. He has malfunctioning of femoral dialysis catheter, central catheter in left internal jugular vein removed a week ago, the right jugulosubclavian territory with thrombosis.

We try cannulation of left internal jugular vein, with ultrasound and radiologic guidance.

During the procedure, the patient experienced sudden and intense chest pain, which disappear spontaneously in a few seconds. At the end of the procedure one of the catheter´s lights was malfunctioning. Before starting hemodialysis the chest pain is reproduced by serum injection. Rx appropriate check. Chest CT is performed and it shown the vascular perforation. The patient suffer mediastinic perforation due to catheter of 14'5F, into the left brachiocephalic vein. The vascular perforation is in the same area of permcath kink previous.

## Discussion

Several mechanical complications associated with central venous catheters, even using ultrasonography and radiology during the procedure, are reported.

It is essential to have a high index of suspicion in patients with known vascular diseases, carriers of thick catheters for long periods, especially left approaches. This creates particular vulnerability to serious complications as vascular perforation.
